# Activation of defence pathways in Scots pine bark after feeding by pine weevil (*Hylobius abietis*)

**DOI:** 10.1186/s12864-015-1546-9

**Published:** 2015-05-06

**Authors:** Andriy Kovalchuk, Tommaso Raffaello, Emad Jaber, Susanna Keriö, Rajendra Ghimire, W Walter Lorenz, Jeffrey FD Dean, Jarmo K Holopainen, Fred O Asiegbu

**Affiliations:** Department of Forest Sciences, University of Helsinki, P.O. Box 27, FIN-00014 Helsinki, Finland; Department of Environmental Science, University of Eastern Finland, P.O. Box 1627, FIN-70211 Kuopio, Finland; Warnell School of Forestry and Natural Resources, The University of Georgia, Athens, GA 30602 USA; Department of Biochemistry, Molecular Biology, Entomology & Plant Pathology, Mississippi State University Mississippi State, Mississippi, MS 397672 USA

**Keywords:** Herbivory, VOC emission, Transcriptomics, Phenylpropanoid pathway, Terpenoid pathway, Protease inhibitors, PR proteins

## Abstract

**Background:**

During their lifetime, conifer trees are exposed to numerous herbivorous insects. To protect themselves against pests, trees have developed a broad repertoire of protective mechanisms. Many of the plant’s defence reactions are activated upon an insect attack, and the underlying regulatory mechanisms are not entirely understood yet, in particular in conifer trees. Here, we present the results of our studies on the transcriptional response and the volatile compounds production of Scots pine (*Pinus sylvestris*) upon the large pine weevil (*Hylobius abietis*) feeding.

**Results:**

Transcriptional response of Scots pine to the weevil attack was investigated using a novel customised 36.4 K *Pinus taeda* microarray. The weevil feeding caused large-scale changes in the pine transcriptome. In total, 774 genes were significantly up-regulated more than 4-fold (p ≤ 0.05), whereas 64 genes were significantly down-regulated more than 4-fold. Among the up-regulated genes, we could identify genes involved in signal perception, signalling pathways, transcriptional regulation, plant hormone homeostasis, secondary metabolism and defence responses. The weevil feeding on stem bark of pine significantly increased the total emission of volatile organic compounds from the undamaged stem bark area. The emission levels of monoterpenes and sesquiterpenes were also increased. Interestingly, we could not observe any correlation between the increased production of the terpenoid compounds and expression levels of the terpene synthase-encoding genes.

**Conclusions:**

The obtained data provide an important insight into the transcriptional response of conifer trees to insect herbivory and illustrate the massive changes in the host transcriptome upon insect attacks. Moreover, many of the induced pathways are common between conifers and angiosperms. The presented results are the first ones obtained by the use of a microarray platform with an extended coverage of pine transcriptome (36.4 K cDNA elements). The platform will further facilitate the identification of resistance markers with the direct relevance for conifer tree breeding.

**Electronic supplementary material:**

The online version of this article (doi:10.1186/s12864-015-1546-9) contains supplementary material, which is available to authorized users.

## Background

Scots pine (*Pinus sylvestris* L.) is one of the most widespread forest tree species in the Northern boreal zone of Eurasia, where its distribution area ranges from the Atlantic coast of Europe in the west to the Pacific coast near the Sea of Okhotsk in the east [1]. It is also cultivated on a large scale and has a major economic importance in the timber, pulp and paper industry. However, insect pests and microbial pathogens pose a serious threat to the extensive monospecific Scots pine plantations. Among them, the large pine weevil (*Hylobius abietis* L., Coleoptera: Curculionidae) is regarded as one of the most important pine pests, causing damage and mortality of young seedlings [2-4]. The weevil breeds predominantly in the bark of roots of felled conifers. It is a typical ‘silvicultural’ pest of plantation forestry, as it occurs at low density in natural habitats. However, high weevil populations develop on the abundant root-stumps left in the ground after clear-cuts [4]. Adult weevils feed on the bark of conifer seedlings during whole summer season, causing high seedling mortality by damaging the bark of the main stem. The adult beetles can live from two to three years. Females lay eggs in June, and feeding is the most active immediately before and at the time of the breeding season. In August, new adult weevils emerge from roots of pine stumps. Together with adults of earlier generation, they feed on plant bark in August – September before moving below ground to hibernate in October [3].

During their lifetime, conifer trees are exposed to numerous herbivorous insects with different feeding strategies and preferences (e.g., bark beetles, weevils, budworms). To protect themselves against the insect attacks, trees have developed a broad arsenal of effective protection mechanisms, including the production of specialised compounds exerting repellent, antinutritive, or toxic effect on herbivores. Moreover, the formation of special anatomical features to store and transport those chemicals, and the synthesis of pathogenesis-related proteins are efficient mechanisms to protect the tree from the herbivore attack [5,6].

Among others, coniferous trees use the oleoresin, a mixture of non-volatile diterpene acids and a large (20-50%) volatile fraction of mono- and sesquiterpenes [7], as a viscose defence tool against damaging herbivores and pathogens. The volatilisation of monoterpenes increases the viscosity of the oleoresin finally leading to the resin polymerisation and the formation of a protective solid plug. Resin-storing conifers constitute an important source of volatile organic compounds (VOCs) mainly dominated by the volatile monoterpenes. In the atmosphere, the conifer VOCs have crucial ecological functions attracting e.g. many herbivorous conifer-feeding species and their natural enemies [8]. In atmospheric processes, the volatile terpenes react with ozone and OH and NO_3_ radicals forming secondary organic aerosols [9,10].

Preformed mechanical barriers and chemical defences are expressed constitutively irrespective of the presence of herbivores, and they provide an efficient protection against many potential invaders. However, upon the perception of an insect attack plants deploy an active defence response at the site of the attack and often systemically throughout the whole plant body [11]. The induced defences are believed to be advantageous for the plant fitness, as they require lower resource allocation costs compared with the constitutive barriers [12,13].

The activation of plant induced defences is a complex biological process that causes massive changes in gene expression throughout the genome [14]. Previous studies have shown that hundreds of genes are either up- or down-regulated in response to the herbivore damage. Several groups of genes have repeatedly been described as induced upon an insect attack, i.e. anti-nutritional proteins (arginases, protease inhibitors, lipoxygenases, peroxidases, polyphenol oxidases and threonine deaminases); potentially toxic proteins (acid phosphatases, chitinases, proteases, hevein-like proteins and leucine aminopeptidases); pathogenesis-related (PR) genes and genes participating in defence-related signalling [15]. Most of the genome-wide transcriptomics studies were performed on angiosperm plants (*Arabidopsis*, tobacco, tomatoes, maize and few others). Transcriptomics studies on conifer trees have been substantially hampered until recently by lacking of their complete genome sequences [14]. Mainly for this reason, there are very few reports describing the transcriptional responses of conifers to insect-induced damages [16,17]. Responses of conifer trees to herbivory have been additionally analysed at the level of proteome, complementing the data available from transcriptomics studies [18]. The scarcity of the available information emphasises the necessity for the further work in this direction. In our experiment, we have combined the microarray-based analysis of changes in the gene expression in Scots pine upon weevil feeding with the analysis of VOC emitted by pine trees. This combined approach should provide better understanding of the underlying mechanisms of the pine’s induced chemical defences and pinpoint the key genes implicated in the defence against herbivores.

## Results

### VOC emission

*Hylobius* feeding significantly increased the total bark VOC emissions (by nearly 2.5-fold) when compared to the intact control plants (Table 1). The total monoterpenes (MT) emissions (20% of the total VOC emissions) were marginally significantly increased (nearly by 3-fold) in the weevil-damaged plants. The emissions of 3-carene (by 6-fold) and limonene (by 7.5-fold) were significantly increased in damaged plants compared to the control. The emissions of total sesquiterpenes (SQTs) were also significantly increased (by 8-fold) as well as were the emissions of several individual SQTs from the weevil-damaged plants compared to the control. Finally, six individual SQTs emitted by the *Hylobius*-damaged seedlings were not detected in the control plants (Table 1).

**Table 1:** **Mean (±SE, n = 7) VOC emission rates measured from the bark surface of the intact control and the** ***Hylobius*** **-damaged Scots pine saplings** ^**a**^

**Emission (ng h** ^**−1**^ **m** ^**−2**^ **(bark area))**	**Control**	**Damage**	***p*** **-value**
Monoterpenes			
α-Pinene^T^	(16.1 ± 9.4)∙10^3^	(10 ± 3.1)∙10^3^	0.597
Camphene^U^	(8.4 ± 5.5)∙10^2^	(8.6 ± 3.4) ∙10^2^	0.674
Sabinene^U^	0 ± 0^b^	(1.7 ± 1.7)∙10^3^	1.000
β-Pinene^U^	0 ± 0^b^	89 ± 73	0.462
3-Carene^T^	(4.9 ± 2.1)∙10^3^	(28.7 ± 12.3)∙10^3^	**0.007**
Limonene^T^	(2.2 ± 1.1)∙10^3^	(15.9 ± 8.1)∙10^3^	**0.013**
β-Phellandrene^U^	84 ± 67	(4.3 ± 3.3)∙10^3^	0.510
1,8-Cineole^U^	0 ± 0^b^	(3.9 ± 2.8)∙10^2^	0.192
Camphor^U^	0 ± 0^b^	(4.7 ± 2.1)∙10^2^	0.070
Bornyl acetate^T^	(4.8 ± 2)∙10^2^	(7.1 ± 1.9)∙10^2^	0.429
* Total monoterpenes* ^*T*^	(24.6 ± 11.7)∙10^3^	(62.7 ± 27.8)∙10^3^	0.060
Sesquiterpenes			
β-Selinene^U^	(2 ± 1)∙10^2^	(3.6 ± 1.1)∙10^2^	0.185
Longicyclene^U^	0 ± 0^b^	(5.8 ± 1.4)∙10^2^	**0.001**
Longifolene^T^	(21.6 ± 7)∙10^2^	(8 ± 2.6)∙10^3^	**0.038**
*trans*-β-caryophyllene^U^	0 ± 0^b^	(5.7 ± 3)∙10^3^	**0.021**
*trans*-β-farnesene^U^	0 ± 0^b^	(21.2 ± 8.4)∙10^2^	**0.021**
Aromadendrene^U^	(2.9 ± 1.4)∙10^2^	(3.4 ± 3)∙10^3^	0.371
α-Muurolene^U^	0 ± 0^b^	(4.1 ± 0.91)∙10^2^	**0.005**
α-Farnesene^U^	0 ± 0^b^	(4.5 ± 2.7)∙10^2^	0.192
δ-Cadinene^U^	0 ± 0^b^	(6 ± 1.8)∙10^2^	**0.021**
*Total sesquiterpenes* ^*T*^	(26.2 ± 8.6)∙10^2^	(21.6 ± 5.9)∙10^3^	**0.001**
Nonanal^T^	(61.4 ± 21.9)∙10^3^	(13.5 ± 2.9)∙10^4^	**0.022**
Methyl salicylate^T^	(50.5 ± 17.7)∙10^3^	(9.7 ± 1.7)∙10^4^	0.082
Total VOCs^T^	(13.9 ± 4.9) ∙10^4^	(31.6 ± 2.6)∙10^4^	**0.008**

^a^Statistical analysis was performed by Independent Sample T-Test (compounds marked with T) and by Mann–Whitney U test (compounds marked with U). ^b^Zero values indicate emission rates below the detection limit. **Emboldened** values indicate statistical significance. VOC emission data was temperature standardized at 30°C.

### Transcriptome response

**Figure 1:**
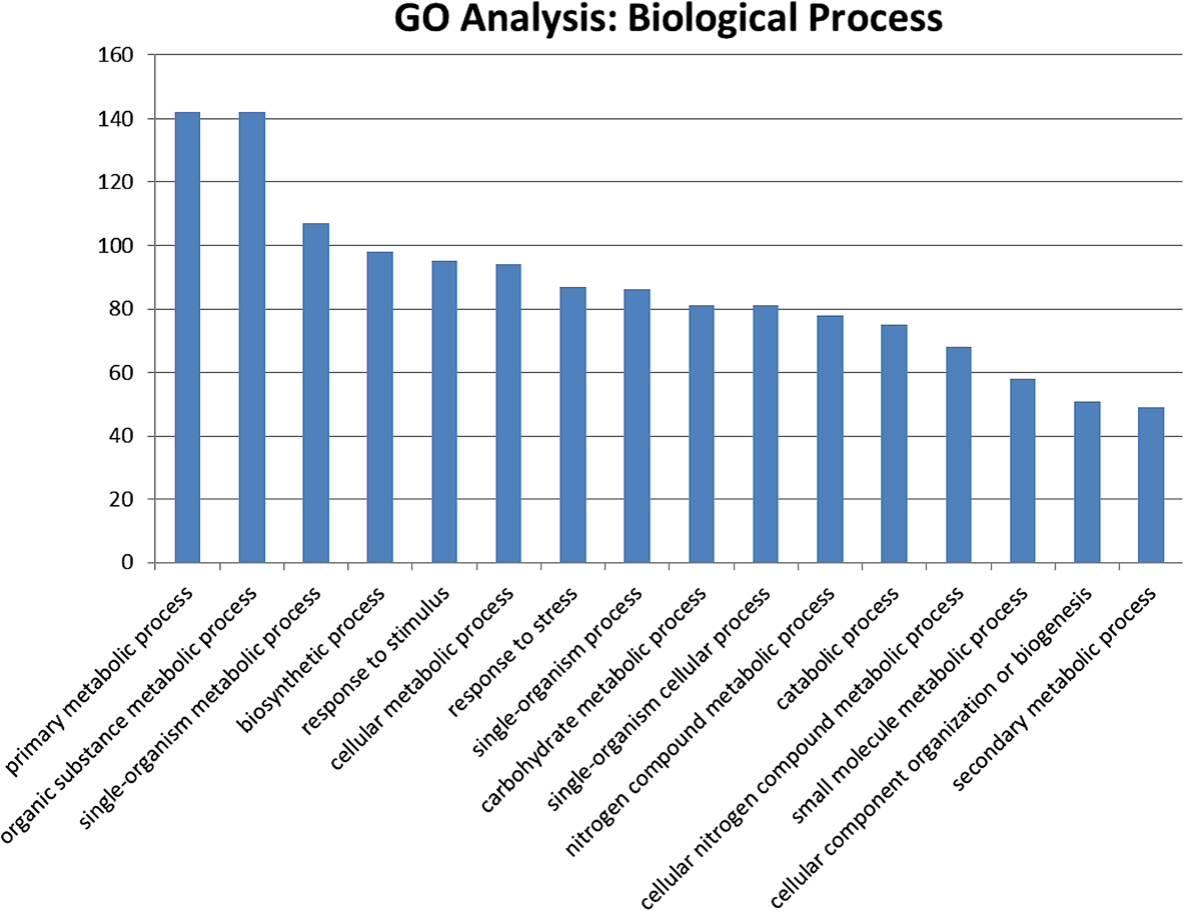
Gene ontology (GO) analysis. Comparison of GO terms from the 774 Scots pine genes significantly induced upon weevil feeding. Biological process GO tags with at least 40 entries per tag are shown.

The weevil feeding-induced damage has caused substantial changes in the pine gene expression. During the initial analysis of the microarray data, we have identified 1581 differentially expressed genes (fold change ≥ 4, *p* ≤ 0.05). Out of those, 1174 genes were up-regulated and 407 genes were down-regulated. However, the adjusted *p* values for all the genes identified in this way were above 0.05. Trying to find a reason for the low statistical support of our results, we noticed that the gene expression pattern in the sample H1 was remarkably different from the two other H samples (H2 and H3) as well as from all three control samples (data not shown). Therefore, we decided to exclude this sample from the further analysis. When the analysis was repeated without taking the sample H1 into account, we identified 838 genes that were significantly differentially expressed (adjusted *p* value ≤ 0.05) (Additional file 1: Table S1), of which 774 genes were up-regulated more than 4-fold, whereas 64 genes were down-regulated more than 4-fold. Out of those, 549 genes (501 up-regulated and 48 down-regulated genes) returned annotation results from Blast2GO (E-value cut off ≤ 1×10^−6^). The distribution of the weevil-induced genes among GO categories indicates large-scale changes in the plant metabolism occurring in response to the insect attack (Figure 1). Additionally, more than 80 genes were associated with the responses to stimuli and/or stress responses.

Twenty-five weevil damage-induced genes with the highest fold change are listed in the Table 2. Among them, putative protease and peptidase inhibitors constitute the most abundant group with 9 representatives, emphasising the role of this class of proteins in the defence against herbivorous insects. On a global scale, many of the pine genes induced by the weevil attack showed a similarity to genes, which are up-regulated in other plant species in response to different types of biotic (e.g., insect or nematode damage, fungal and bacterial infections) and abiotic stresses (wounding, hyperosmotic stress, high salt stress, water deprivation etc.). Based on the similarity to the *Arabidopsis* genes, they were classified in several functional groups. Some of those groups (e.g., secondary metabolism, transcriptional regulation, signalling and pathogenesis-related genes) were represented by a high number of transcripts that will be discussed in more details below.

**Table 2:** **Twenty five pine genes most highly up-regulated by weevil feeding-induced damage**

**SEQ_ID** ^**a**^	**log2_FC** ^**b**^	**Adj.** ***p*** **-value**	**Hit name** ^**c**^	**Hit description** ^**c**^	**E-VALUE**
isotig14898	9.174	0.0034	AT1G73260	trypsin and protease inhibitor family protein/Kunitz family protein	2.00E-17
isotig08882	8.797	0.0038	AT1G72060	serine-type endopeptidase inhibitor	6.00E-06
isotig08888	8.748	0.0093	AT1G72060	serine-type endopeptidase inhibitor	5.00E-06
isotig14897	8.707	0.0034	AT1G73260	trypsin and protease inhibitor family protein/Kunitz family protein	2.00E-17
isotig11956	8.584	0.0034	AT1G73260	trypsin and protease inhibitor family protein/Kunitz family protein	3.00E-15
isotig15076	8.530	0.0038	AT5G05390	LAC12 (laccase 12); laccase	0
isotig08893	8.349	0.0041	AT1G72060	serine-type endopeptidase inhibitor	2.00E-06
isotig08890	8.341	0.0038	AT1G72060	serine-type endopeptidase inhibitor	3.00E-06
isotig00382	7.941	0.0042	No hit		
isotig00365	7.904	0.0038	No hit		
isotig14893	7.894	0.0083	AT1G73260	trypsin and protease inhibitor family protein/Kunitz family protein	2.00E-17
isotig27556	7.741	0.0050	AT2G45220	pectinesterase family protein	1.00E-125
isotig25461	7.710	0.0085	AT3G01420	ALPHA-DOX1, DOX1, DIOX1; lipoxygenase	1.00E-100
isotig08884	7.666	0.0034	AT1G72060	serine-type endopeptidase inhibitor	9.00E-08
isotig08898	7.602	0.0058	AT1G75620	glyoxal oxidase-related	1.00E-149
isotig35240	7.558	0.0069	AT1G14190	glucose-methanol-choline (GMC) oxidoreductase family protein	1.00E-128
isotig13645	7.541	0.0038	AT1G08080	ATACA7, ACA7 (ALPHA CARBONIC ANHYDRASE 7)	3.00E-59
isotig00361	7.493	0.0050	No hit		
isotig19954	7.452	0.0093	AT3G22400	LOX5; electron carrier/ iron ion binding/lipoxygenase/metal ion binding/oxidoreductase	0
isotig13655	7.427	0.0051	AT4G16260	catalytic/ cation binding/hydrolase, hydrolyzing O-glycosyl compounds	3.00E-78
isotig00384	7.332	0.0136	No hit		
isotig15012	7.268	0.0041	AT1G64160	disease resistance-responsive family protein/dirigent family protein	7.00E-47
isotig15009	7.250	0.0094	AT1G64160	disease resistance-responsive family protein/dirigent family protein	7.00E-47
isotig41906	7.234	0.0091	AT2G21050	amino acid permease, putative	1.00E-118
isotig40293	7.127	0.0058	AT4G23340	oxidoreductase, 2OG-Fe(II) oxygenase family protein	2.00E-42

^a^Seq_IDs correspond to the names of the sequences in the PtNewbler1 assembly available from the Conifer DBMagic database [47]. ^b^Binary logarithm of the fold change value. ^c^Correspond to the best hit of BLASTX searches against The Arabidopsis Information Resources (TAIR) database.

### Genes with a role in signal perception and signalling pathways

Perception of pests and pest-induced damage is of a vital importance for the development of induced plant defence responses. In plants, the signal perception is mainly performed by different classes of the leucine-rich repeat (LRR) receptors, either membrane-anchored or soluble ones. The LRR receptors can activate signalling cascades via a physical interaction with protein kinases or, alternatively, they might contain their own kinase domain. In our experiment, we have observed up-regulation of 5 transcripts showing similarity to LRR receptors and 13 genes encoding putative LRR receptor-like kinases. It should be noted, however, that a number of the predicted LRR receptors (7 isotigs) and LRR receptor-like kinases (4 isotigs) were down-regulated upon the weevil feeding (Additional file 1: Table S1).

Signalling molecules (e.g., jasmonic acid (JA), salicylic acid (SA) and ethylene) play a crucial role in the regulation of plant responses to biotic and abiotic stresses. Among them, JA and ethylene are the key players in the formation of plant response to wounding and insect-induced damage. In our experiment, we have observed the induction of several genes with a known role in the octadecanoid pathway, a biochemical route used by plants to produce JA and methyl jasmonate (MeJA): allene oxide synthase (3 isotigs), 12-oxophytodienoate (OPDA) reductase (4 isotigs) and putative OPDA-CoA ligase (1 isotig). Additionally, genes showing similarity to *A. thaliana* DAF1, the positive activator of chloroplastic phospholipase A1 expression, and to *A. thaliana* WR3, the nitrate transporter involved in the JA-dependent signal transduction, were also up-regulated in response to the weevil feeding compared to the control. However, we have also observed an increased expression level of several genes that are known either to attenuate the jasmonate signalling cascade or to negatively control the expression of JA-regulated genes, i.e. the cytochrome CYP94B3 functioning as jasmonoyl-isoleucine-12-hydroxylase and thus reducing the level of JA-Ile (3 isotigs), and JAZ proteins JAZ1 (2 isotigs), JAZ2 (3 isotigs), JAZ9 (1 isotig), JAZ10 (3 isotigs) and JAZ12 (3 isotigs). Some of these genes have been previously reported to be induced by wounding and/or by a fungal infection. Their activation might also be a part of a negative feedback control pathway.

The weevil feeding caused also the induction of a gene with similarity to acetyl CoA:(Z)-3-hexen-1-ol acetyltransferase (CHAT), an enzyme catalysing the formation of (Z)-3-hexen-1-yl acetate [19]. This compound is the major volatile released upon mechanical wounding or herbivore damage of green leaves as well as conifer needles [20,21]. It induces plant defence reactions and may also participate in the plant-to-plant signalling, but its functional role in conifers received very little attention so far.

### Genes involved in transcriptional regulation

The activation of signalling pathways eventually results in the transcriptional induction of certain target genes. It is usually achieved via activation of the specific transcription factors. The weevil-induced damage resulted in the up-regulation of a number of genes encoding predicted transcription factors (TFs) compared to the undamaged control. These TFs showed similarity to the families ERF (15 isotigs), bHLH (7 isotigs), NAC (6 isotigs), MYB (6 isotigs), WRKY (5 isotigs) and bZIP (2 isotigs) (Additional file 1: Table S1). Members of these groups have been reported to play an important role in plant responses to biotic and abiotic stresses [22-25].

### Genes involved in the regulation of plant hormone homeostasis

Several genes with a potential function in the regulation of the plant hormone homeostasis were up-regulated in our study. Among them, we can name 9-cis-epoxycarotenoid dioxygenase, a key enzyme in the biosynthesis of abscisic acid (ABA) (2 isotigs); two auxin UDP-glycosyltransferases (5 isotigs), two indole-3-acetic acid (IAA)-amido synthases (6 isotigs), two predicted auxin transporters (2 isotigs), gibberellic acid (GA) methyltransferase (1 isotig), a predicted gibberellin receptor (1 isotig) and genes with similarity to two *Arabidopsis* proteins involved in GA signalling, SLEEPY1 and LBD40 (3 and 1 isotig, respectively) (Additional file 1: Table S1).

### Secondary metabolism genes

**Figure 2:**
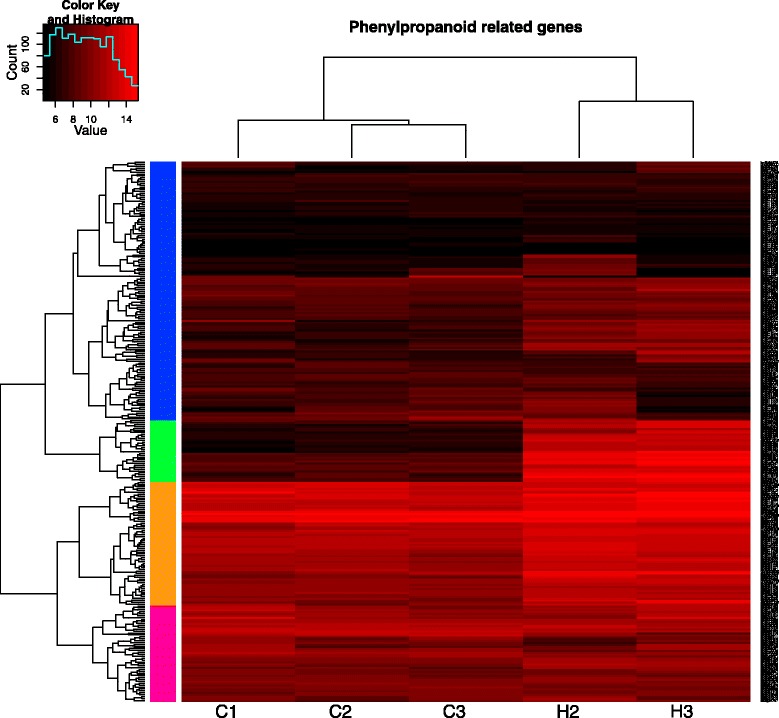
Cluster analysis of genes involved in phenylpropanoid pathway. The scheme illustrates expression values of the genes with a predicted role in phenylpropanoid pathway as well as predicted genes encoding laccases and dirigent proteins (based on the BLASTX hits of the corresponding isotigs). Colours on the scheme correspond to the binary logarithms of the expression values of the depicted genes; genes shown in black have the lowest expression levels, whereas genes shown in bright-red have the highest expression values.

The phenylpropanoid pathway occupies a central position in the plant defence reactions. In addition to its primary function, the supply of precursors for the lignin biosynthesis, it produces a number of important metabolites, e.g. flavonoids, anthocyanins, stilbenes, condensed tannins and phenolics. Our results demonstrate the transcriptional induction of multiple genes involved not only in the phenylpropanoid pathway itself, but also in the upstream shikimate and phenylalanine biosynthesis pathways (Figure 2). The up-regulated genes showed similarity to the bifunctional 3-dehydroquinate dehydratase/shikimate dehydrogenase (isotig14787), arogenate dehydratase (6 isotigs), phenylalanine ammonia lyase (PAL) (contig57512), 4-coumarate-CoA ligase (isotig11247 and isotig28403), cinnamyl alcohol dehydrogenase (isotig18966) and cinnamoyl-CoA reductase (isotig08777 and isotig08778). We have also observed induction of the genes encoding putative chalcone synthases (5 isotigs), pinoresinol reductase (3 isotigs) and two groups of genes with a predicted role in the flavonoid biosynthesis, which are members of the CYP75B1 subfamily of cytochromes P450 (2 isotigs) and UDP-glucose glycosyltransferase (9 isotigs). Interestingly, 3 isotigs showing a similarity to KFB20, the negative regulator of phenylpropanoid pathway that targets PAL for the degradation, were also up-regulated in our experiments. The induction of these genes might indicate the activation of the negative feedback loop controlling the first step of the phenylpropanoid pathway.

**Figure 3:**
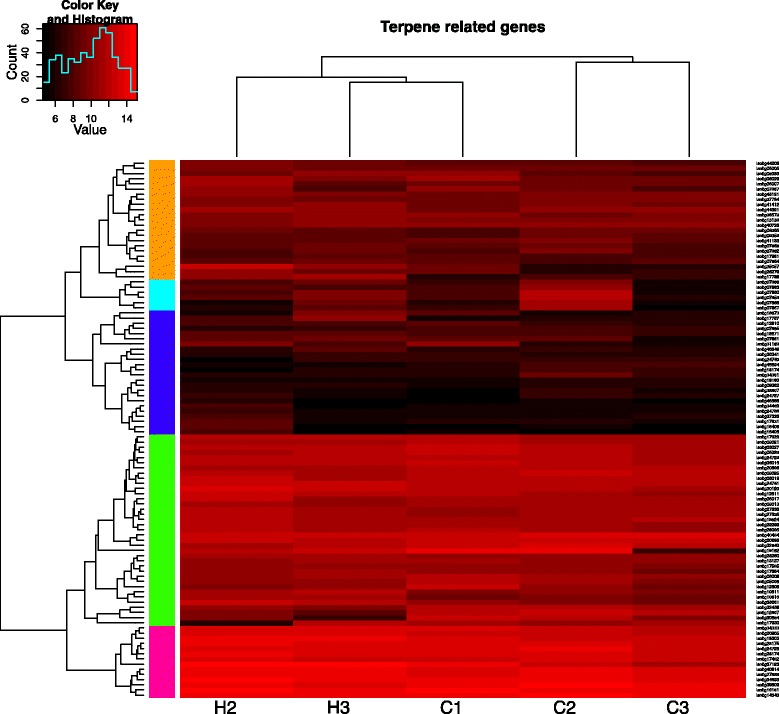
Cluster analysis of genes involved in terpenoid pathway. The scheme illustrates expression values of the genes with a predicted role in terpenoid biosynthesis pathway (based on the BLASTX hits of the corresponding isotigs). Colours on the scheme correspond to the binary logarithms of the expression values of the depicted genes; genes shown in black have the lowest expression levels, whereas genes shown in bright-red have the highest expression values.

Terpenoids also play an essential role in the constitutive and induced chemical defence of conifer trees against pathogens and herbivores. However in our experiment, the expression of genes involved in the terpenoid biosynthesis pathway remained largely unaffected by the weevil feeding (Figure 3). The only gene that was significantly induced (isotig17788) encodes a predicted terpene synthase. It shows the highest similarity to the dual function (*E,E*)-α-farnesene synthase/(*E*)-β-ocimene synthase from interior spruce (*Picea englemannii* x *Picea glauca*) [26] and to the farnesene synthase from Norway spruce (*Picea abies*) [27].

### Cell wall reinforcement

The formation of lignin from phenylpropanoid pathway-derived monolignols is mediated by the combined action of several classes of proteins. It is generally accepted that both laccases and class III peroxidases are involved in the monolignol oxidation and radicalisation [28], whereas dirigent proteins are believed to control the radical-radical coupling [29-31]. In our experiment, we have observed the massive induction of all the three classes of genes involved in the lignin formation. In total, 20 isotigs showing similarity to the class III peroxidase genes, 30 putative laccase isotigs and 10 isotigs with similarity to the dirigent genes were strongly up-regulated in response to the weevil feeding (Additional file 1: Table S1). Additionally, a number of genes with a potential role in the cell wall remodelling were induced by the weevil-caused damage, including genes with a similarity to pectin methylesterases (14 isotigs) and uclacyanin (3 isotigs).

### Defence-related genes

This group encompasses genes encoding proteins presumably implicated in the active defence against herbivores and pathogens. Many of them are known as PR (pathogenesis-related) proteins, but we also included here some unclassified proteins with a potential role in defence reactions. One of the most prominent classes of the defence-related genes in our analysis was represented by protease inhibitors. In total, we identified 18 genes up-regulated by the weevil herbivory and showing similarity to various types of protease inhibitors (the Kunitz-type and potato type II serine proteinase inhibitors; cysteine proteinase inhibitors). Remarkably, 9 out of the 15 genes showing the highest fold change in our experiment were represented by the predicted protease inhibitor-encoding genes (Table 2). We also observed the induction of genes encoding putative β-1,3-glucanases (15 isotigs) and three different classes of chitinases: class III (2 isotigs), class IV (11 isotigs) and class V (2 isotigs). Other PR genes up-regulated in response to the weevil damage include genes encoding thaumatin- and osmotin-like proteins (family PR-5; 3 isotigs), lipid-transfer proteins (family PR-14; 6 isotigs) and germin-like proteins (family PR-16; 3 isotigs) (Additional file 1: Table S1). Genes belonging to the family PR-9 (‘lignin-forming peroxidases’) were massively induced in our experiment, and they have been discussed earlier together with other proteins contributing to the cell wall reinforcement. We have also observed the up-regulation of several genes that might have their primary role in defence reactions, but are not formally classified yet as the PR genes, e.g. genes showing similarity to the *A. thaliana* acid phosphatase with anti-insect activity (At5g24770) [32], to the *Arabidopsis* heat-stable protein with antimicrobial activity (At3g17210) [33] or to a putative pathogenesis-related protein (At3g19690) (Additional file 1: Table S1). Several other induced genes might contribute to the plant defence in different ways. The cysteine peptidase (1 isotig) may disrupt the peritrophic membrane protecting insect gut epithelium, whereas lipoxygenase (LOX) (3 isotigs) may covalently modify dietary proteins [6].

### Validation of microarray results with qPCR

**Figure 4:**
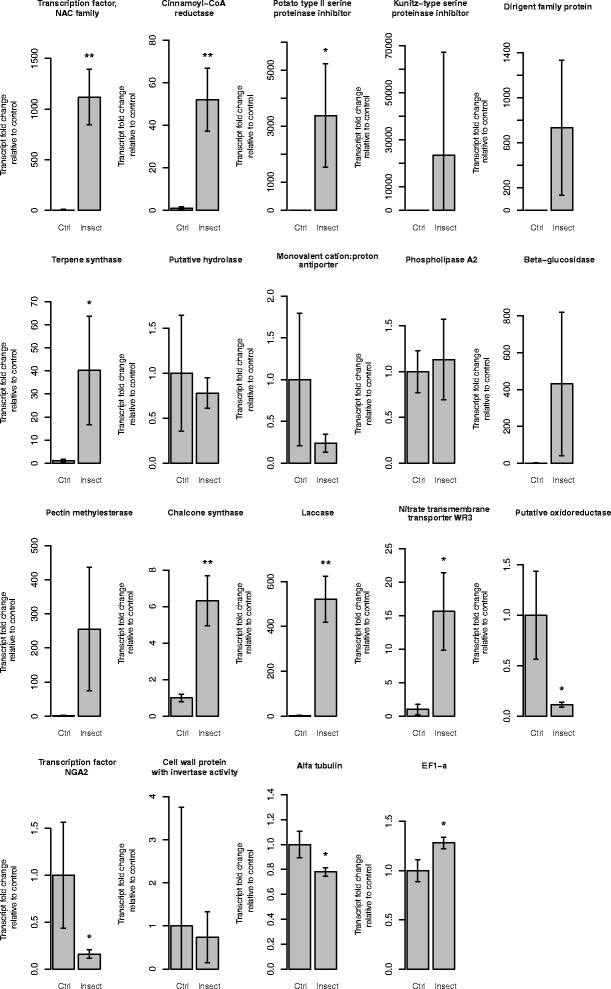
qPCR analysis of 17 selected genes and 2 reference genes. Shown data are based on two technical replicates. Error bars represent standard deviations. Genes marked with asterisks show statistically significant differences in expression level: a single asterisk (*) indicates genes with *p* value 0.1 < *p* < 0.5, and double asterisk (**) indicate genes with *p* value *p* ≤ 0.1. Predicted functions of the genes are based on their BLASTX hits with known function.

In order to evaluate the reliability of the microarray data, we designed gene-specific primers and performed qPCR analysis for 17 genes. The genes were selected based on their expression pattern (up- or down-regulated), high fold change and potential biological significance. As a control, we have used two reference genes, α-tubulin and elongation factor EF1-a. Overall, the results of the qPCR experiment were in good agreement with the microarray results (Figure 4). However, three genes (isotig28679, isotig23410 and contig51269) showed no significant changes in expression in the qPCR experiment, whereas their differential expression in the microarray experiment was statistically significant. Additionally, in several cases the observed gene expression fold change was higher in the qPCR experiment than it could be deduced from the microarray data. The cross-hybridisation between closely related genes of the same gene family might be one of the factors affecting the results of the microarray analysis.

## Discussion

The transcriptomic responses of plants against herbivores were extensively studied on several model species of angiosperms, in particular on *Arabidopsis*, tobacco, tomato and maize [15]. However, to our best knowledge, only two reports describing the transcriptional response of conifer trees to insect attacks have been published so far [16,17]. Both of the previous works used spruce species as their experimental models. Results of our experiments, therefore, provide a first insight into the intrinsic mechanisms of the defence reactions against herbivorous insects in such important forest tree species as Scots pine. The coverage of the microarray platform used in our study (36.4 K cDNA elements) significantly exceeds the coverage of the platforms used for spruce previously (9.7 K and 21.8 K, respectively). The better coverage should allow the detection of novel defence-relevant genes that might have escaped their identification in the earlier experiments. Furthermore, the high correlation of transcript level for the same tissues between *P. sylvestris* and *P. taeda* (r = 0.93) [34] permitted differential screening to be done using the loblolly pine arrays with RNA obtained from Scots pine.

The gene expression pattern in one of the weevil-damaged saplings (H1) was considerably different from the pattern observed in the five remaining plants. This was the main reason to exclude this sample from further analysis. We do not have a definite explanation for the deviating pattern of the sample H1. This could be a result of technical error during sample processing or hybridisation, but we also cannot exclude that it was due to genotype-specific differences between the plants used in our experiments.

The results obtained in this study clearly show the large-scale changes in the pine transcriptome upon the weevil feeding. Many of the identified genes have been previously demonstrated to be induced upon insect attack in other model species. In particular, components of the phenylpropanoid pathway, the JA biosynthesis and signalling pathways, numerous transcription factors, genes involved in the cell wall reinforcement and several types of protease inhibitors were strongly up-regulated. The simultaneous induction of a high number of genes emphasises that the defence against herbivores is a highly complex process involving numerous metabolic and signalling pathways and thus requiring a high degree of coordination between them. In particular, the up-regulation of genes encoding diverse groups of transcriptional factors reflects the massive rearrangements in the host plant’s transcriptional profile in response to the insect attack and correlates with the need for the rapid simultaneous induction of hundreds of genes.

Plants have the ability to detect herbivorous insect attacks via perceiving the so-called herbivore-associated molecule patterns (HAMPs). The HAMPs can originate from plant cell components modified as a result of the herbivore-produced damage or, alternatively, they can be represented by the insect-specific elicitors, e.g. derived from insect oral secretions. Regardless the nature of the HAMPs, their recognition is achieved via binding with the specialised receptor proteins. Most of plant receptors belong to the so-called leucine-rich repeat (LRR) type receptors. We have observed induction of a number of genes encoding predicted LRR receptors and LRR receptor-like kinases in our experiment. At the same time, several genes of this group were down-regulated. The observed repression might be caused by insect-derived effectors, as it has been proposed recently that herbivorous insects are capable of partly supressing the defence genes of their host plants [35].

The jasmonate pathway has a dominant role in regulating the plant defence reactions in response to insect herbivory [6]. Our data have also demonstrated the induction of several components of the JA biosynthesis and signalling pathways upon the weevil feeding. However, we could also observe the up-regulation of a number of genes that might be a part of a negative feedback control over the JA signalling. Some of them were previously reported to be induced by the wounding, and their induction might be a part of a mechanisms controlling the intensity of plant’s defence response.

Plant secondary metabolites play a central role in the constitutive and induced chemical defence against herbivores. In our experiment, we have documented a massive induction of genes involved in the different branches of the phenylpropanoid pathway, as well as into some upstream steps. This finding once again emphasises the central role of this pathway in the plant defence response. Also, numerous genes involved in the conversion of monolignols into lignin polymer, i.e. class III peroxidases, laccases and dirigent proteins, were strongly up-regulated. At the same time, the transcriptional response of the terpenoid pathway remained rather weak, as only a single predicted terpene synthase was significantly induced. This is rather unexpected as a resin flow was observed in the damaged area. It might be partly explained by the fact that the resin in the beetle-wounded bark area of the attacked pine is primarily transported from the constitutive resin storage in other parts of the canal system [36]. Furthermore, the availability of VOC data only from the earlier time point in the growing season does not provide sufficient information for any definitive conclusions on the relationship between the transcriptional control of terpene biosynthesis in the twig phloem and the terpene emission. We cannot exclude that the differences in the physiological stage of the saplings at the time points of the VOC emission analysis and the sampling for the transcriptome analysis (end of June – beginning of July and end of August – beginning of September, respectively) affected the results of those experiments, making more difficult a direct comparison between them.

Earlier experiments with conifer seedlings [37,38] have demonstrated that the VOC production in the needles is induced stronger than in the stem base where the *H. abietis* damage occurred. This observation suggests that in young pine trees the synthesis of terpenes might occur predominantly in the photosynthesising tissues, followed by the fluid resins allocation to the constitutive resin storage close to the damaged area. If this suggestion is correct, our sampling strategy may have biased the microarray results, as we have isolated RNA only from the phloem of the damaged twigs, and not from the needles. Alternatively, the regulation of enzymes of the terpene biosynthesis in pine might predominantly occur at the different level, e.g. at the level of translation or post-translationally. Interestingly, in another experiment the changes in the pine terpenoid pathway upon fungal infection were less pronounced as compared with spruce [17]. However, additional experiments will be required to figure out whether there are some fundamental differences in the regulation of the terpenoid biosynthesis between these two genera of conifer trees.

We observed that the weevil herbivory induced several classes of the defence-related proteins. Among them, the protease inhibitors occupied an outstanding position due to the high fold change in their expression levels. Remarkably, 9 out of the 15 most highly up-regulated genes encode predicted protease inhibitors. This observation is in line with previous reports on the important role of protease inhibitors in the defence against herbivores [6,11]. It is assumed that they affect the insect’s digestive physiology by inhibiting gut proteases [6]. Other up-regulated defensive proteins might affect the insect digestion system in a different way, e.g. cysteine protease was reported to disrupt the peritrophic membrane of insect gut epithelium, whereas lipoxygenases can modify dietary proteins reducing their nutrition value.

It has been reported that the pine weevil feeding on stem bark of Scots pine seedlings increased the emission of monoterpenes and sesquiterpenes from damaged bark by nearly 4-fold and 7-fold, respectively [37], whereas our results showed an increase in the emission of MTs and SQTs by 3-fold and 8-fold, respectively, from the healthy bark, just below the damaged area. This might be an indication of a systemic response of the terpene synthesis to the bark damage which was earlier reported as the increased terpenoid emission from needles of the *Hylobius*-damaged pine [37] and Norway spruce [38] saplings. Additionally, several SQTs (i.e., longicyclene, *trans*-β-caryophyllene, *trans*-β-farnesene, α-muurolene, and δ-cadinene), which remained below the detection limit in the control group, were emitted by the weevil-damaged plants. The emission of *trans*-β-farnesene has repeatedly been shown to be induced by insect herbivory or oviposition [39-41], and this compound is also known to attract parasitoid and predatory insects [42]. Thus, the weevil herbivory in our experiment has resulted not only in quantitative, but also in qualitative changes in the spectrum of compounds emitted by pine saplings. It was partly unexpected that we did not find a clear transcriptional response in the terpene biosynthesis-related genes in the weevil damaged area. It would be interesting to investigate the specificity of the only terpene synthase gene (isotig17788) significantly induced in our experiment to address the question of the correlation between its induction and changes in the emission spectrum. The gene shows the highest similarity to the dual function (*E,E*)-α-farnesene synthase/(*E*)-β-ocimene synthase from interior spruce (*Picea englemannii* x *Picea glauca*) [26]; however, the deduced amino acid sequence of the protein encoded by isotig17788 is only 86% identical to the sequences of the previously characterised enzyme, and it is known that even few amino acid changes can dramatically alter the product spectrum of a terpene synthase [43]. Taking together, these observations suggest that in conifer seedlings the majority of terpenes released from the damaged tissue are synthesised elsewhere and transported to the damage site.

In our experiment, we have observed the induction of a putative gene for acetyl CoA:(Z)-3-hexen-1-ol acetyltransferase (CHAT), an enzyme catalysing the formation of (Z)-3-hexen-1-yl acetate. However, we could not detect the presence of this compound in our VOC samples. It is possible that the compound is predominantly emitted by needles, as has been demonstrated before [20,21], whereas its levels in bark emissions remained below the detection level.

The presented results provide an important insight into the defence mechanisms employed by Scots pine to counteract insect attacks. This study will improve our understanding of the defence reactions in conifer trees and provide a framework for new pest control strategies. The data can also be used for the identification of new resistance marker of potential importance in tree breeding.

## Conclusions

In this work, we present the results of the GC-MS analysis of induced pine-emitted volatiles and the analysis of the pine transcriptional response to the insect herbivory. Weevil-induced damage resulted in massive increase of VOC emissions by pine saplings. Not only the amount of emitted volatiles, but also their composition was influenced by insect herbivory.

Our data show an extensive similarity between the responses to herbivory in pine, spruce and flowering plants. Many of the genes identified in our experiment have been previously shown to be induced upon herbivore attack in other plant species, and their biological role is well-understood. At the same time, numerous up-regulated genes could not be annotated due to their low similarity to the known proteins, and those are particularly interesting as they might represent novel, previously uncharacterised components of the pine’s defence machinery. Additional experimental efforts will be required in order to elucidate their biological role.

The obtained data are important for the large-scale comparative analysis of transcriptional responses to the herbivory in conifers and flowering plants. The improved 36.4 K pine microarray used in this work represents a significant advancement over the microarrays used in previous studies on conifer trees. The better coverage allows for the identification of many novel hits of potential interest. The presented data will improve our understanding of the defence reactions of conifer trees. They are also of importance for the development of novel markers for the breeding of tree cultivars with the improved resistance against herbivores.

## Methods

### Seedling information

We used six-years-old, approximately 1 m high Scots pine (*Pinus sylvestris* L., Pinaceae) saplings obtained from a commercial nursery (Taimityllilä Oy, Mäntyharju, Finland). The seedlings were individually planted in 7.5 L plastic pots in a 2:1 (v/v) quartz sand (0.5-1.2 mm diameter, SP Minerals, Partek, Finland) and fertilised *Sphagnum* peat (Kekkilä PP6, Finland) mix. Seedlings were fertilised with Taimiston kestolannos (N 9%, P 5%, K 5%, Mg 5%, S 4% and micronutrients, Kemira Oy, Finland). The potted plants were grown in a field site at the University of Eastern Finland (UEF) Research Garden with natural rainfall and supplementary watering (if required). The same seedling provenance has been used for the herbivore-induced VOC analyses in previous years by [20] and [37].

### Herbivory treatment and RNA sampling

The study site was Kuopio campus research garden of the UEF (62°53′N, 27°37′E, and 80 m above sea level). We randomly selected three Scots pine saplings for the damage treatment group (H-plants) and the same number of seedlings for the control treatment group (C-plants). Six *H. abietis* beetles were kept in the base of the two lowest whorl branches (three beetles per branch were enclosed inside a 12 cm × 12 cm mesh cloth cage with plastic foam frames) of all H-plants. *Hylobius* weevils (enclosed into cage) continued feeding on the plants for eight days (August 25 – September 2, 2011). The same-sized empty cages were also attached to the adjacent branches of the C-plants to create similar environmental conditions.

For the microarray analysis, bases of the lowest whorl branches from the H- and the C-plants were collected and put into liquid nitrogen at −78°C on September 2, 2011. The samples were preserved in ultra-low freezer (−80°C) racks/boxes until they were used for the RNA extraction (see below). The heights of all the H and the C plants were measured before the plants were harvested.

### Herbivory treatment for VOC sampling

We randomly selected seven Scots pine saplings for the *Hylobius*-damaged treatment and seven for the control treatment. The feeding on pine stem by *Hylobius* beetles started on 28 June and continued until the VOC sampling was done on July 6, 2011. We reported VOC results from this trial as actual VOC samples of RNA sampling in the later growing season were lost due to technical problems in the GC-MS system. The feeding period of this trial (8 days) by *Hylobius* beetles was the same as in the RNA experiment and similar feeding cages were used, but fixed on the main stem. Same-sized empty cages were also attached to the C plants to create similar environmental conditions. *Hylobius*-damaged bark surface area was more or less the same in both of the experiments, although four beetles per plant were used in the VOC experiment and six beetles were used during the RNA experiment later in the growing season.

### VOC sampling and analysis

The VOC samples were collected from the stem bark surface of both the control and the *Hylobius* damaged seedlings by enclosing them in cleaned polyethylene terephthalate (PET) bags (heated at 120°C for 1 h) (size 25 × 38 cm, FREETIME, Suomen Kerta Oy, Finland). The stem section just below the *Hylobius*-damaged bark area was enclosed by the cleaned PET bag in such a way that the length of the bag was at the right angle to the direction of the stem and the open end of the bag was sealed with a duct tape to make it air-tight. Clean charcoal-filtered and MnO_2_-scrubbed air was pumped into the bags via Teflon tubing at a rate of 0.6 L/min to flush the system for 10 minutes. The VOC sample was pulled through to steel tubes filled with 150 mg of Tenax TA-adsorbent (Supelco, mesh 60/80, Perkin Elmer) at a rate of 0.2 L/min through an opening cut at the outermost corner of the bag. We collected 4.5 L of VOC samples from all the control and the damaged saplings. The VOC sampling was done using the pump-operated VOC collection system designed for the field work [20]. The temperature inside the PET bags was monitored by wireless temperature/humidity loggers (Hygrochron DS1923-F5 i Button, Maxim Integrated Products, Inc., CA). During the VOC sampling, the average temperature inside the collection bags was 31°C. After the VOC sampling, the radius (r) and the length (l) of the VOC-sampled stem section was measured and the stem bark surface area (A) was calculated using the equation A = 2π∙r∙l. We measured the *Hylobius*-damaged bark surface area (average 12 cm^2^) which was approximately 13% of the total surface area of the VOC-sampled stem section (average 91 cm^2^).

The VOC samples were analysed by gas chromatography–mass spectrometry (GC-MS) (Hewlett Packard GC type 6890, MSD 5973, Beaconsfield, UK) as described by [20]. Different VOCs were identified by comparing their mass spectra with the Wiley library and pure standards. The compounds lacking standards were quantified manually using α-pinene for MTs and longifolene for SQTs assuming that the responses would be the same as the responses of the standards. The authentic standards for camphene, β-pinene, 3-carene, camphor, nonanal and methyl salicylate were purchased from Aldrich, for α-pinene, 1,8-cineole, longifolene, trans-β-farnesene, aromadendrene and δ-cadinene from Fluka, and for limonene, bornyl acetate and trans-β-caryophyllene from Sigma.

The detection limit set in the chromatograms was 1 ng, and the concentrations of 1 μl of injected standards were between 35 and 50 ng. Standard emission rates were calculated at 30°C using the temperature-dependent algorithm [44]. We used the temperature coefficient (β) of 0.09 for MTs [44] and of 0.18 for SQTs [45] to standardize the emissions.

VOC emissions were calculated in ng h^−1^ m^−2^ (bark area) using the following equation:

$$ E=\frac{F\left(C2-C1\right)}{A} $$

\Where E = VOC emissions, F = flow rate of input air (l/h), C2 = concentration of compound per litre volume of output air (ng/l), C1 = concentration of compound in input air (considered as 0 ng/l) and A = bark surface area (m^2^). Analysis of VOC emission data was performed with IBM SPSS Statistics 19 for Windows (International Business Machines Corp. Armonk, New York, US).

### cDNA preparation for microarray experiments

Samples from the three pine branches damaged by *Hylobius* feeding and the three pine control branches were harvested and frozen in liquid nitrogen. The phloem of each sample was collected separately, followed by RNA extraction. RNA was extracted from the samples as described elsewhere [46].

The RNA samples from each sample were processed as follows: 1 μg of total RNA was treated with DNase I (Promega, Finland), and incubated for 30 min at 37°C followed by the DNase I inactivation at 65°C for 10 min. The treated RNA was then purified using RNeasy MinElute Cleanup Kit according to the manufacturer's instruction (Qiagen, Finland) and eluted in 14 μl nuclease-free water. RNA quality and integrity were assessed using Agilent RNA 6000 Nano Kit and Agilent 2100 Bioanalyzer following the manufacturer's instruction (Agilent Technologies, Germany). Total RNA (100 ng) was subjected to reverse transcription and amplification using the whole transcriptome amplification (WTA) kit according to the manufacturer's instruction (Sigma-Aldrich, Finland). In order to avoid transcript abundance alteration in the sample, the minimal number of 17 amplification cycles was used. The cDNA generated with the WTA kit was purified with the GenElute PCR Clean-Up kit (Sigma-Aldrich, Finland) and eluted in 50 μl nuclease-free water. The cDNA was run on 1.5% agarose gel to assess the integrity and the range of fragment length obtained after the amplification with the WTA kit.

### Microarray hybridisation and analysis

In this work, we have made use of a novel customised *Pinus taeda* microarray. The microarray was designed based on *P. taeda* transcriptome assembly PtNewbler1 available from the Conifer DBMagic database [47]. The assembly is derived from five *P. taeda* cDNA libraries prepared using multiple tissues from multiple genotypes. Details of the libraries construction, sequencing and assembly are described elsewhere [47]. The sequence selection and probe design for the microarray were performed by NimbleGen. The microarray composed of 109,272 probes (three probes per gene model). Microarray data can be accessed at the NCBI Gene Expression Omnibus (GEO) under accession number GPL19078. 4 μg of cDNA from each sample was sent to NimbleGen (Roche, Iceland) for the hybridisation on the *P. taeda* customised microarray. The cDNA hybridisation was carried out at NimbleGen facilities (NimbleGen, Iceland) according to their standard protocols. All the required equipment, reagents and procedures were provided and executed by NimbleGen.

The raw data were analysed in R software [48] using the open source software for bioinformatics *Bioconductor* [49]. In particular, the *oligo* package was used to create the microarray annotation and to normalise the raw data [50]. The *limma, genefilter,* and *gplots* packages were used for the cluster analysis and to apply statistical tests to the normalised data in order to retrieve statistically significant differentially expressed genes [50-52]. After a linear model was fit to the microarray data, a moderated t-test, moderated F-statistic, and log-odds of differential expression by empirical Bayes moderation of the standard errors towards a common value were calculated. The *p*-values were adjusted by fdr methods. The data discussed in this publication have been deposited in NCBI's Gene Expression Omnibus [53] and are accessible through GEO Series accession number GSE60383.

### Microarray data validation by quantitative PCR

A selected number of genes which were differentially expressed in the microarray data were validated by standard quantitative PCR (qPCR). The selected genes and the information related to the specific primers are summarised in Additional file 2: Table S2. Primers were designed based on loblolly pine sequences from the PtNewbler1 assembly [47], and they were confirmed in Scots pine by comparison to unpublished raw transcriptome sequences (Prof. Teemu Teeri, personal communication). The cDNA used for the microarray hybridisation was first diluted 10-fold. After the dilution step, each reaction was performed as follow: 5.5 μl of cDNA as template (62.2 ng < cDNA < 70.4 ng), 7.5 μl LightCycler 480 SYBR Green Master Mix (Roche, Finland), and 1 μl of forward and reverse primers (0.7 μM final concentration). Each reaction was run in 2 technical replicates. The qPCR was performed in a Roche LightCycler 480II machine (Roche, Finland) with the following program: pre-incubation at 95°C for 5 min and 45 amplification cycles (95°C for 10 sec, 60°C for 10 sec, and 72°C for 10 sec). A final melting curve analysis was also included to assess the primer specificity. The crossing points (Cp) values were calculated with the 2nd derivative method using the Roche LightCycler 480 software (version 1.5.1.62). Two reference genes, elongation factor 1 alfa (*EF1-α*, [54]) and alfa tubulin (*TUBA* [55]), were used to normalise the data. Finally, the Cp values were imported and analysed in R software [48] using the *EasyqpcR* package [56].

### Availability of supporting data

The data set supporting the results of this article is included within the article and its additional files.

## Additional files

Additional file 1: Table S1. Complete list of pine genes differentially expressed upon weevil attack. Additional file 2: Table S2. List of the primers used in the qRT-PCR analysis.
